# Relationship between sleep quality, mood state, and performance of elite air-rifle shooters

**DOI:** 10.1186/s13102-022-00424-2

**Published:** 2022-02-25

**Authors:** Jiaojiao Lu, Yan An, Jun Qiu

**Affiliations:** Shanghai Research Institute of Sports Science (Shanghai Anti-Doping Agency), Shanghai, China

**Keywords:** Competition performance, Elite air-rifle shooters, Mood state, Sleep quality

## Abstract

**Background:**

To evaluate the impact of pre-competition sleep quality on the mood and performance of elite air-rifle shooters.

**Methods:**

Elite shooters who participated in an air-rifle shooting-competition from April 2019 to October 2019 were evaluated using actigraphy, including Total Sleep Time (TST), Sleep Efficiency (SE), Sleep Latency (SL), Wake-time after Sleep Onset (WASO). Sleep quality was assessed by Pittsburgh sleep quality index (PSQI) and Profile of Mood State (POMS). Mood state was assessed by Competitive State Anxiety Inventory-2.

**Results:**

Study included 23 shooters, of them 13 male and 10 female with the mean age 23.11 ± 4.82 years. The average time to fall asleep was 20.6 ± 14.9 min, TST was 7.0 ± 0.8 h and SE was 85.9 ± 5.3%. Average sleep quality was 5.2 ± 2.2 and tended to decrease as the competition progressed. Pre-competition sleep time in female athletes was significantly higher compared to the competition day (*P* = 0.05). Pre-competition SL was significantly longer in women than in men (*P* = 0.021). During training and pre-competition, the tension, fatigue, depression, and emotional disturbance were significantly lower in athletes with good sleep quality. Athletes with good sleep quality had significantly more energy. The PSQI total score positively correlated with cognitive anxiety (r = 0.471, *P* < 0.01), and somatic anxiety (r = 0.585, *P* < 0.01), and negatively correlated with energy (− 0.504, *P* < 0.01) and self-confidence scores (r = − 0.523, *P* < 0.01).

**Conclusion:**

Poor sleep quality negatively impacted the mood of athletes; however, sleep indices and competition performance of athletes during competitions were not significantly correlated.

## Background

Air-rifle shooting is a precision sport and has complex performance requirements. Research has shown that good sleep quality is important for better physical and emotional recovery of athletes, which, in turn, ensures excellent performance [1]. Shooters not only have to maintain their concentration during long-term training and competitions but also need to have quick reactions during each shot and deal with tension and pressure for long periods of time during major competitions. These demanding sports consume more energy and often cause sleep problems, such as difficulty falling asleep and waking up at night, especially before major competitions [2, 3].

Sleep and circadian rhythms have a direct relationship with cognitive and metabolic functions [4]. In sports science, sleep time and sleep quality are considered to be the key factors affecting athletic ability, recovery after exercise, and sports performance [5]. Recently, an increasing number of studies have investigated the relationship between emotional and mental health and the performance of athletes during competitions [6–9]. Good sleep plays an important role in the sports performance, energy recovery, disease damage control, metabolism, cognitive memory, and emotional health of elite athletes, allowing the ability for better physical and emotional recovery [10]. Although competition-related stress might interfere with the sleep patterns, it was reported that discipline and focused training for highly competitive sports have positive effects on the sleep quality of professional athletes [11]. Therefore, sleep monitoring and regulation have become important aspects of the pre-competition preparation and regimen of athletes.

The science and technology team in-charge of the Shanghai shooting team found that the motor pattern, training content, competition level, and mood state of elite athletes had an impact on their sleep[12]. This study aimed to evaluate the impact of pre-competition sleep quality on the mood and performance of elite air-rifle shooters. We hypothesized that not only stress but other factors, common for professional athletes, influence relationship between pre-competition sleep quality, mood and sports performance. Obtained data may be used for establishing effective pre-competition sleep evaluation and regulation protocols to ensure high performance and well-being of athletes.

## Methods

### Subjects

We conducted a descriptive study on the Shanghai elite air-rifle shooting team. Data were collected from April to October 2019 during the preparation period for the national competition. The inclusion criteria were as follows: (1) elite air-rifle shooters at the national level or above, (2) systematic training, and (3) consistent training time and training program. The exclusion criteria involved retired athletes and athletes taking medication for medical conditions, including sleep medication. This study was approved by the Scientific Research Ethics Committee of Shanghai Research Institute of Sports Science (Shanghai Anti-Doping Agency), and written informed consents were obtained from all participants.

### Data collection and definition

Data including age and gender as well as sleep diary information were collected from at least two completed national competitions. Sleep quality was evaluated using the Pittsburgh sleep quality index (PSQI) scale [13], mood state was evaluated using the Profile of Mood State (POMS) scale [14], and competition performance was evaluated by recording the number of rings.

According to the training arrangement, the research was divided into four stages: baseline, pre-competition, competition-day, and post-competition. The baseline stage represented daily training. The daily training time was 5.5 ± 1.5 h and included live firing training exercises. The number of shells spent was 116.9 ± 8.5. The pre-competition stage included the three days before the competition: usually the day before departure, registration day, and pre-competition training day. The competition-day stage included the day of the competition, and the post-competition stage referred to the day after the completion of the final event by the athlete.

The Objective sleep index was recorded during all the above-mentioned stages using Actigraph GT3X+ (Actigraph LLC, Version 6.13.4) which was worn around the non-dominant wrist, and the collected data were analyzed using the Actilife 6.13 software [15]. The Actigraph GT3X+ is a small wearable device which has been proved to be able to obtain highly consistent information in combination with polysomnography, and is widely used in research to monitor the sleep of elite athletes [16, 17], and based on the principle of three-axis accelerometer and algorithm technology [18]

The sleep quality data were obtained from the PSQI questionnaire and a sleep diary. The Cole-Kripke algorithm [19] was used to automatically obtain the sleep-related indices of the athletes’ sleep/wake behavior, including Total Sleep Time (TST), Sleep Latency (SL), Wake-time after Sleep Onset (WASO, determined by the awakening frequency and duration), and Sleep Efficiency (SE). The Actigraph counts were generated and sleep consistency was evaluated. The following conditions were set during data collection: (1) if the Actigraph was not worn according to the instructions, data interruption could be clearly recorded on the Actigraph, and the data not fulfilling the requirements would not be considered; (2) if the sleep time difference between the data from the sleep diary and Actigraph was > 30 min, the sleep data would be adjusted to complete the data of the Actigraph; and (3) the sleep diary would be used to correct sleep latency.

The PSQI scale, which was used to evaluate subjective sleep quality, consists of 19 separate items that generate a total of seven component scores: subjective sleep quality, sleep latency, sleep persistence, habitual sleep efficiency, sleep disorders, use of sleep medications, and daytime dysfunction [13]. The overall score reflected the subjects' sleep quality over the previous month, with a score of 5 or higher considered a sign of poor sleep quality [20]. According to the Guidelines for the Diagnosis and Treatment of Insomnia for Chinese Adults [21], abnormal sleep signs indicated nighttime sleep time of < 7 h, nighttime sleep latency of > 30 min, nighttime awakening, nightmares, daytime sleepiness, and poor sleep quality. Recording information in the sleep diary was mainly used to correct sleep latency. The average value of five consecutive daily training days was considered as the baseline sleep time. The data of the pre-competition stage was based on the average value of two of the three major national competitions in the entire year. The sleep data of the athletes participating in the qualifying and eliminating rounds of the 10-m air-rifle competition were collected on the day of the competition.

A short version of the POMS scale was revised by Zhu Beili et al. in 1995. It is a concise alternative and brief but accurate measure of the mood of athletes. The POMS-short form [14] and the Chinese norm were used to evaluate the mood of the athletes at the various stages. There are 40 questions in the short form of POMS and each answer is scored on a scale of 0–4. Finally, the scores of five negative emotions, namely, tension, anger, fatigue, depression, and panic and two positive emotions, namely, energy and self-regard were obtained. The Competitive State Anxiety Inventory-2 (CSAI-2) questionnaire [22] compiled by Martens et al. and revised by Zhu Beili et al. in 1994 and the Chinese norm were used to evaluate the competitive state of the athletes. The 27-item questionnaire is divided into three subscales: cognitive anxiety, somatic anxiety, and self-confidence. Each subscale was scored separately on a scale of 1–4. A high score indicated high cognitive anxiety, somatic anxiety, and self-confidence. Race scores were recorded from the total ring value of the 10-m air rifle from the national shooting competition.

### Statistical analysis

SPSS 22.0 software (IBM, USA) was used for statistical analysis, and the measured data were expressed as mean ± standard deviation. The intra-group analysis of variance was used to compare the sleep quality of athletes at the baseline, pre-competition, competition-day, and post-competition stages. The stage of the competition was used as the intra-group factor (a total of seven grades: baseline; pre-competition days 3, 2, and 1; competition days 1 and 2; and post-competition day 1) in each analysis of variance. An independent samples *t* test was used to analyze the differences in sleep score indices measured by Actigraphy and mood state between different genders. Pearson’s correlation coefficient (r) was used to analyze the correlation between sports performance, sleep quality, and mood state. *P* < 0.05 was considered to be statistically significant.

## Results

Twenty-three elite air-rifle athletes were enrolled in our study (13 men and 10 women; average age, 23.11 ± 4.82 years). Four athletes had competed internationally and all had > 2 years of experience at the national level.

**Fig. 1:**
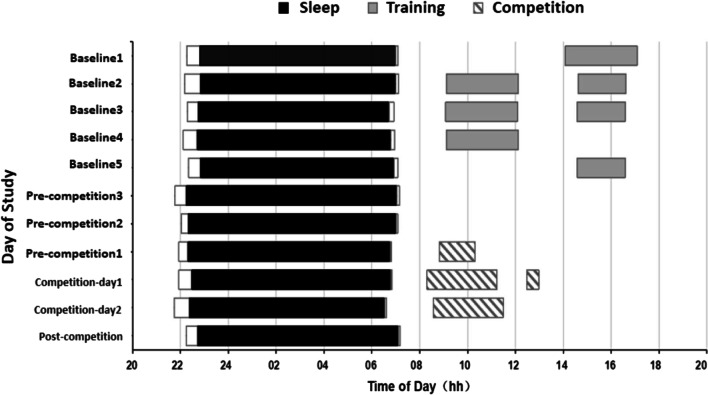
Sleep/wake behavior of 23 shooters at the baseline and before, during, and after the competition. Each line represents a 24-h period from 20:00 to 20:00. The first five lines represent the sleep/wake behavior during baseline. The remaining lines represent the 3 pre-match days, 2 nights preceding and following the match day, and 1 day after the match. The black and white horizontal bars represent the average of all nighttime sleep times, including sleep time, sleep latency, start of sleep, and wake time. The light grey horizontal bar indicates the training time at baseline. The black and white bar indicates the time of the match

It took the athletes 20.6 ± 14.9 min to fall asleep. TST was 7.0 ± 0.8 h, sleep efficiency was 85.9 ± 5.3%, subjective sleep quality was 5.2 ± 2.2, and there was no obvious difference between bedtime and waking-up time during the training and competition stages (Fig. 1 and Table 1). Sleep-onset time, total time in bed, TST, SE, and WASO changed during the different stages. Sleep-onset time was the most delayed at baseline and on the day after the competition. Sleep-onset times on pre-competition day 2 and the first day of the competition were significantly earlier than that on the day after the competition (*P* = 0.030, *P* = 0.049; Table 1). There was no significant change in SL. The total time in bed on the day after the competition was significantly lower than that on pre-competition day 3 and the day 1 of the competition (*P* = 0.047, *P* = 0.026; Table 1). The TST decreased as the competition progressed, and the TST on the day after the competition significantly decreased compared to that at the baseline stage and on the pre-competition day 3 (*P* = 0.021, *P* = 0.045; Table 1). SE was the highest at the baseline stage. Compared to that at baseline, SE significantly decreased on the first day of the competition and the day after the competition (*P* = 0.035, *P* = 0.017; Table 1). WASO was the longest on the day after the competition, which significantly differed from that at the baseline and on the three days before the competition (*P* = 0.040, *P* = 0.023; Table 1). The awakening frequency did not change at different stages. The subjective sleep quality significantly changed between the competition and non-competition periods. The subjective PSQI score in the pre-competition stage was higher than that at baseline (*P* = 0.12; Table 1), indicating that the athletes believed that the sleep quality was regular during the competition.

**Table 1:** Sleep index variables at the baseline, pre-competition, competition day, and post-competition stages ($\overline{x}$ ± *s*)

Sleep index variables	Baseline		Pre-competition		Competition day		Post-competition	
	Male (n = 13)	Female (n = 10)	Male (n = 13)	Female (n = 10)	Male (n = 13)	Female (n = 10)	Male (n = 13)	Female (n = 10)
Sleep latency (min)	12.1 ± 8.9	14.7 ± 10.7	20.2 ± 4.6^#^	26.6 ± 7.2*^#^	25.5 ± 12.1^#^	26.7 ± 17.1^#^	11.3 ± 8.3	6.8 ± 4.9*
Total sleep time (h)	7.3 ± 0.4	7.9 ± 0.9	7.4 ± 0.9	7.2 ± 0.9^#^	7.4 ± 0.8	7.0 ± 0.9^#^	6.1 ± 0.8^#^	6.6 ± 0.5^#^
Sleep Efficiency (%)	91.3 ± 3.2	88.1 ± 3.7	86.6 ± 3.3	84.8 ± 7.9	81.9 ± 4.2^#^	85.3 ± 5.0	78.5 ± 7.3^#^	81.3 ± 3.1^#^
WASO (min)	38.6 ± 9.3	67.3 ± 15.1	57.6 ± .6.1	69.3 ± 11.9	85.3 ± 10.3	78.8 ± 19.0	103.9 ± 25.5	92.3 ± 18.9
PISQ > 5	7.3 ± 1.3		8.1 ± 1.5					
PISQ ≤ 5	3.6 ± 1.6		4.3 ± 0.9					

^#^Significant difference. Mean values with the same superscript are significantly different (*P* < 0.05); *P < 0.05 compared with opposite gender

**Fig. 2:**
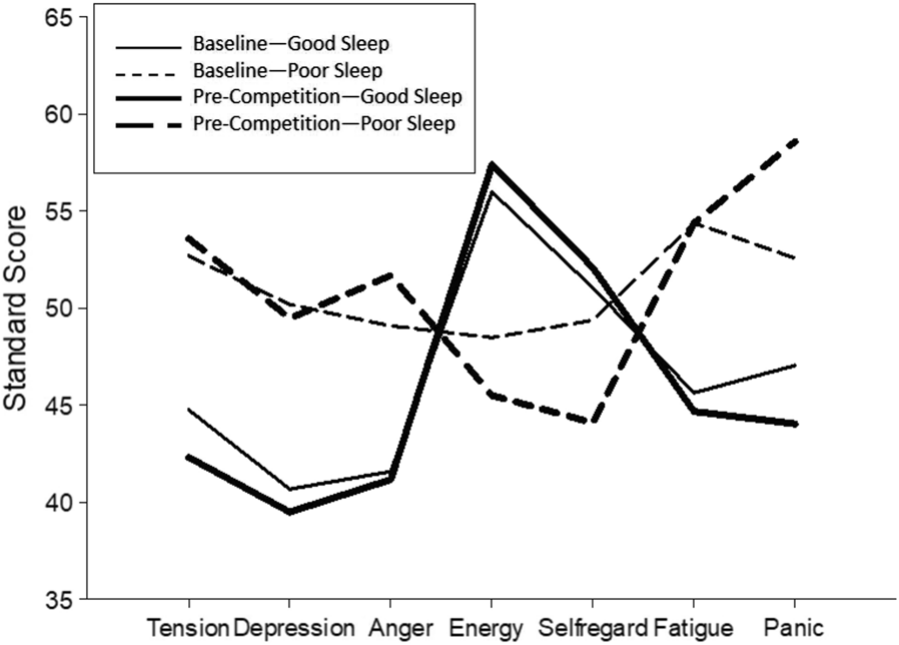
Comparison of the mood states of athletes with different sleep quality

During the daily training stage, athletes with good sleep quality exhibited significantly lower tension (*P* = 0.018), fatigue (*P* = 0.026), depression (*P* = 0.039), and total of emotional disturb (TMD) (*P* = 0.004) scores than athletes with poor sleep quality. Consequently, athletes with good sleep quality had significantly higher energy (*P* = 0.045) than those with poor sleep quality. Before the competition, tension (*P* = 0.002), anger (*P* = 0.009), fatigue (*P* = 0.007), depression (*P* = 0.011), panic (*P* = 0.000), and TMD (*P* = 0.000) of athletes with good sleep quality were significantly lower than those of athletes with poor sleep quality. Energy (*P* = 0.001) and self-regard (*P* = 0.039) were significantly higher in athletes with good sleep quality than in those with poor sleep quality (Fig. 2). The curves of athletes with good sleep in the baseline and pre-competition stages are iceberg shaped while the curves of athletes with poor sleep are inverted iceberg shaped, which directly reflects the differences in the overall emotional levels of athletes with different sleep qualities (Fig. 2).

At baseline, overall subjective sleep quality (PSQI) score was 5.2 ± 2.2, and there were 7 people with PSQI > 5 (average PSQI score of 7.3 ± 1.3), accounting for 30.4% of the total number. In the pre-competition stage, there were 8 participants whose PSQI > 5 (average PSQI score 8.1 ± 1.5), accounting for 34.8% of the total number of participants. The total PSQI score was positively correlated with the POMS negative emotion subscale, TMD, cognitive anxiety, and somatic anxiety and negatively correlated with the POMS positive emotion subscale (energy and self-regard) and self-confidence (all *P* < 0.05; Table 2). The sleep quality, time of falling asleep, sleep disorders, and daytime function on the PSQI scale were positively correlated with the POMS negative emotion subscale, TMD, cognitive anxiety, and somatic anxiety and negatively correlated with energy and self-confidence (all *P* < 0.05; Table 2). Sleep quality was negatively correlated with daytime function and self-regard (all *P* < 0.05; Table 2). Sleep time was positively correlated with only fatigue (*P* < 0.05; Table 2). We then analyzed the correlation between the PSQI scores, sleep quality, sleep time, total sleep time, sleep efficiency, sleep disorders, daytime function, and competition performance. Our results revealed no significant correlation between competition performance and sleep indices (all *P* > 0.05; Table 3).

**Table 2:** Correlation analysis (r) between sleep index variables and mood state variables one week before the competition

Mood states variables (r)	PSQI score	Sleep quality	Sleep time	Total sleep time	Sleep efficiency	Sleep disorders	Daytime function
Tension	0.616**	0.487**	0.520**	0.208	0.130	0.589**	0.448**
Anger	0.515**	0.444**	0.446**	0.124	0.009	0.471**	0.444**
Fatigue	0.523**	0.480**	0.300*	0.286*	0.213	0.300*	0.493**
Depression	0.613**	0.547**	0.511**	0.152	0.117	0.547**	0.474**
Energy	− 0.504**	− 0.470**	− 0.337*	− 0.111	− 0.098	− 0.307*	− 0.595**
Panic	0.598**	0.411**	0.455**	0.197	0.123	0.628**	0.521**
Self-regard	− 0.284*	− 0.328*	− 0.143	− 0.056	− 0.113	− 0.142	− 0.327*
TMD	0.669**	0.585**	0.500**	0.206	0.148	0.534**	0.605**
Cognitive anxiety	0.471**	0.390**	0.418**	0.175	0.165	0.502**	0.204
Somatic anxiety	0.585**	0.524**	0.512**	0.191	0.001	0.562**	0.426**
Confidence	− 0.523**	− 0.436**	− 0.351**	− 0.181	− 0.153	− 0.465**	− 0.471**

Significant correlation between sleep index variables and mood state variables

*P < 0.05;**P < 0.01

**Table 3:** Correlation analysis between sleep index variables and race scores in the competition

Sleep index variables (Mean ± SD)		Score (number of rings) (Mean ± SD)	*r*	*P*
PSQI score	6.23 ± 1.18	623.1 ± 3.45	0.308	0.555
Sleep quality	0.86 ± 0.67		0.316	0.562
Sleep time	0.28 ± 0.45		0.398	0.631
Total sleep time	7.3 ± 0.9		0.404	0.291
Sleep efficiency	87.2 ± 5.4		0.248	0.538
Sleep disorders	0.89 ± 0.58		0.320	0.319
Daytime function	1.51 ± 0.83		0.366	0.555

A correlation analysis was conducted between athletic performance and scores of POMS indices and three CSAI-2 scores. The results showed a statistically significant negative correlation between competition scores and depression subscale (*P* = 0.002), and a significant negative correlation was observed between competition scores and somatic anxiety in the CSAI-2 scale scores (*P* = 0.025; Table 4).

**Table 4:** Correlation analysis between mood state variables and number of rings in competition

Mood state variables (Mean ± SD)			Race scores (number of rings) (Mean ± SD)	*r*	*P*
POMS	Tension	47.26 ± 9.28	623.1 ± 3.45	− 0.127	0.092
	Anger	43.98 ± 10.59		− 0.091	0.079
	Fatigue	48.84 ± 10.61		− 0.221	0.052
	Depression	43.82 ± 9.4		**−** 0.738**	**0.002**
	Energy	53.71 ± 10.24		0.215	0.067
	Panic	48.78 ± 7.66		0.019	0.141
	Self-regard	50.36 ± 9.79		0.122	0.098
	TMD	97.28 ± 19.28		0.009	0.179
CSAI-2	Cognitive anxiety	20.17 ± 8.54		0.182	0.070
	Somatic anxiety	17.33 ± 5.61		**−** 0.674*	**0.025**
	Confidence	21.00 ± 7.4		0.191	0.083

*P < 0.05; **P < 0.01 significant correlation with number of rings in competition

## Discussion

In this study, we analyzed the acute effect of sleep quality on athletes’ mood and performance through subjective sleep evaluation and objective monitoring data. The main findings showed that poor sleep quality of athletes has a negative impact on the mood; however, there was no significant correlation between sleep indices and competition performance of athletes during competitions. Our results suggested that sleep changes under competition stress in athletes, but studies should focus more on sleep quality than on duration. The pre-competition preparation protocols and the time required for post-competition recovery should be adjusted and effective training plans should be formulated to ensure that athletes receive proper rest and recovery.

We observed that the daily sleep of shooters was consistent and the sleep quality was acceptable; however, their sleep/wake cycles were affected by competition stress. This was more evident among the female athletes. Only subjective sleep quality changed during the competition and non-competition periods. The TST before, during, and after the competition was lower than that during the daily training stage. The SL, SE, and WASO had negative changes to varying degrees, and SL before and after the competition significantly increased. The decrease in SE is mainly caused by WASO. Our results are consistent with those of previous studies [23, 24], which suggested that athletes’ sleep changes under the influence of competition stress, but more attention should be paid to sleep quality than to sleep duration. In addition, in events that span over a longer duration, this change in sleep pattern manifests throughout the competition and negatively affects subsequent training after the competition. On further analyzing the self-rated sleep pattern before the competition, we found that the severity of sleep problems before the competition was greater than normal and the distribution of PSQI scores was remarkably high (the higher the score, the worse the sleep quality), indicating that most athletes experienced lack of energy before the competition. Although the minimum recommended sleep time for adults to prevent health damage and decreased work efficiency is approximately 7 h [25], it may not be enough for athletes. Studies have shown that persistent sleep deprivation can negatively impact the body's motor performance, such as endurance, speed and muscle strength, as well as cognitive performance, such as attention, skill performance and the ability to learn; it was recommended for elite athletes to sleep longer, due to the increased amount of training and energy consumption during competitions [26–28]. Furthermore, sleep patterns may differ between genders due to the physiological and psychological differences between men and women. There is a growing body of evidence that suggests a correlation between the gut microbiome and sleep quality [29]. In-depth research and investigation should be conducted on this topic. Individual sleep analysis of elite athletes revealed that the total time in bed in the daily training stage is usually not lower than that in the competition stage. The bedtimes of many athletes in the daily training stage is earlier than that in the competition stage, but this is not reflected in the TST and sleep efficiency. This is because athletes usually do not fall asleep immediately after going to bed but may indulge in activities that stimulate the brain, which hampers effective sleep time and quality [30]. Although athletes consciously go to bed earlier than usual in the competition stage, different sleep rhythms and poor sleep consistency may lead to longer SL, increased sleep fragmentation, and decreased sleep quality.

The investigation of the mood state of athletes revealed that the pre-competition mood of male athletes was better than that of the female athletes. Therefore, gender should be considered as a factor affecting the mood state of athletes before a competition. On comparing the mood state of athletes at different sleep levels, we observed that sleep quality was directly related to mood. PSQI scores of ≤ 5 and > 5 represent good sleep quality and poor sleep quality, respectively. Our results showed that athletes with different sleep qualities in the baseline and pre-competition stages showed the same results, i.e., the athletes with good sleep quality had significantly lower negative emotions, higher scores of positive emotions, and lower TMD than those with poor sleep quality. Figure 2 shows that athletes with good sleep presented iceberg-shaped curves, while those with poor sleep presented inverted iceberg-shaped curves. These results directly reflect the differences in mood state and are consistent with the results of several previous studies [31, 32]. Lack of quality sleep will lead to negative mood changes, which negatively affects the athletes’ cognition, decision-making skills, and motor skills [33]. Moreover, poor sleep quality and lack of sleep increase fatigue and tension in athletes, affecting their success in competitions. Therefore, it is very important for athletes to have good sleep, which will contribute to effective physical and emotional recovery. However, we found that sleep time is correlated only with fatigue and has little correlation with emotion. Therefore, the TST of athletes seems to be not the main factor affecting their emotional state.

According to the results of the correlation analysis of sports performance, sleep, and emotion, we did not find any relationship between pre-competition sleep quality and sports performance. In fact, many studies have found that despite the variation in sleep patterns of athletes during the competition, it does not always lead to poor performance [26, 34]. However, our study showed that better sports performance is indicative of lower depression and somatic anxiety level of athletes. In addition, this finding is consistent with that of previous studies, showing that emotion has a predictive effect on sports performance [35, 36]. Although there is no direct relationship between sleep and sports performance, the high correlation between sleep and emotion indicates that sleep may affect sports performance through the interaction between sleep and emotion. The findings of this study illustrate three points: first, sleep is not the main factor affecting sports performance; second, it is necessary to investigate whether change in sleep is caused by emotional factors or location changes during competitions; and lastly, long-term changes in sleep are concerning and need to be investigated. Previous studies have proved that long-term decline in sleep quality can lead to an imbalance in the autonomic nervous system, resulting in athletes experiencing symptoms similar to those experienced during overtraining, resulting in a decline in immunity and cognitive function [37, 38]. A single episode of sleep deprivation was found to affect the glutamine content in rats, resulting in a decline of the body’s ability to exercise [39] Although coaches and athletes believe that sleep is a part of the recovery process, sleep quality is often not considered in the training plan and competition protocol [40]. Due to the lack of attention on the importance of sleep training, in some cases, sleep time is sacrificed by increasing time for physical training or other activities. When athletes experience sleep problems, most of them have no corresponding strategies to improve their poor sleep. Therefore, sleep training should focus on enhancing the sleep management of athletes, such as improving healthy sleeping habits, ensuring sleep consistency, finding the causes of sleep problems, and regularly evaluating and receiving feedback on the sleep quality of athletes. These processes will help athletes maintain good sleep, reduce the interaction between sleep and emotions, and in turn, improve sports performance.

This study has certain limitations. Since professional athletes' training content, training load intensity, training years, competition experience and competition level will all have an impact on sleep and mood, we cannot expand the sample size to select athletes who have almost the same training content, daily training duration, and ammunition use before the same game. In addition, each type of competitive sport has its own training characteristics, thus our findings should be tested in a study with a large number of participants.

## Conclusions

Our study demonstrated that sleep and mood are factors that influence the well-being of athletes during competitions, therefore, more attention should be paid to the sleep quality and mood state of athletes before, during, and after high-performance and precision sport competitions. The impact of improper sleep on performance may accumulate over time, leading to negative changes in the emotional state of athletes by affecting their mood, which in turn affects sports performance. Taking our data into consideration can help coaches and trainers better understand the implications of such changes in athletes, adjust pre-competition preparation protocols, and the time required for post-competition recovery, and formulate effective training plans to ensure that athletes receive proper rest and recovery.

## Data Availability

The datasets used and/or analysed during the current study are available from the corresponding author on reasonable request.

## Abbreviations

PSQI: Pittsburgh sleep quality index
POMS: Profile of Mood State
CSAI-2: Competitive State Anxiety Inventory-2
